# HOW DO HEALTH AND MARITAL STATUS SHOCKS AFFECT DISPARITIES AT OLDER AGES?

**DOI:** 10.1093/geroni/igae098.0971

**Published:** 2024-12-31

**Authors:** Richard Johnson

**Affiliations:** Urban Institute, Washington, District of Columbia, United States

## Abstract

This paper measures the incidence of health and marital shocks after age 65, assesses their impact on household wealth, and compares outcomes by race/ethnicity and other characteristics. The study focuses on the need for long-term services and support (LTSS), help with everyday activities that can be provided at home by unpaid family caregivers or paid helpers, or in residential care settings or nursing homes. Using data from the Health and Retirement Study spanning 1995 to 2020, the analysis measures how substantial LTSS needs, chronic health conditions, LTSS use, widowhood, and divorce affect the level of and changes in household wealth and the probability that older adults exhaust most of their resources. We find that health and marital shocks are common at older ages. After age 65, 70 percent of adults develop substantial LTSS needs, 28 percent enter a nursing home, 51 percent develop a major medical condition, and 29 percent become widowed. The lifetime risk of developing substantial LTSS needs or a major medical condition does not vary much by race/ethnicity. However, Black and Hispanic adults generally experience these shocks at younger ages than non-Hispanic white adults. Black adults are more likely to become widowed than others. Older adults who develop substantial LTSS needs, and especially those who enter a nursing home, experience much sharper declines in household wealth than other older adults. The financial impact of the onset of LTSS needs does not appear to vary much by race/ethnicity. Other shocks have smaller financial impacts.

